# Linden (*Tilia cordata*) associated bumble bee mortality: Metabolomic analysis of nectar and bee muscle

**DOI:** 10.1371/journal.pone.0218406

**Published:** 2019-07-10

**Authors:** Claire Lande, Sujaya Rao, Jeffrey T. Morré, Gracie Galindo, Julie Kirby, Patrick N. Reardon, Gerd Bobe, Jan Frederik Stevens

**Affiliations:** 1 Department of Crop and Soil Science, Oregon State University, Corvallis, Oregon, United States of America; 2 Department of Chemistry, Oregon State University, Corvallis, Oregon, United States of America; 3 NMR Facility, Oregon State University, Corvallis, Oregon, United States of America; 4 Linus Pauling Institute, Oregon State University, Corvallis, Oregon, United States of America; 5 Department of Animal and Rangeland Sciences, Oregon State University, Corvallis, Oregon, United States of America; 6 Department of Pharmaceutical Sciences, Oregon State University, Corvallis, Oregon, United States of America; University of California San Diego, UNITED STATES

## Abstract

Linden (*Tilia* spp.), a profusely flowering temperate tree that provides bees with vital pollen and nectar, has been associated with bumble bee (*Bombus* spp.) mortality in Europe and North America. Bee deaths have been attributed, with inadequate evidence, to toxicity from mannose in nectar or starvation due to low nectar in late blooming linden. Here, we investigated both factors via untargeted metabolomic analyses of nectar from five *T*. *cordata* trees beneath which crawling/dead bumble bees (*B*. *vosnesenskii*) were observed, and of thoracic muscle of 28 healthy foraging and 29 crawling bees collected from linden trees on cool mornings (< 30°C). Nectar contained the pyridine alkaloid trigonelline, a weak acetylcholinesterase inhibitor, but no mannose. Principal component analysis of muscle metabolites produced distinct clustering of healthy and crawling bees, with significant differences (*P*<0.05) in 34 of 123 identified metabolites. Of these, TCA (Krebs) cycle intermediates were strongly represented (pathway analysis; *P*<0.01), suggesting that the central metabolism is affected in crawling bees. Hence, we propose the following explanation: when ambient temperature is low, bees with energy deficit are unable to maintain the thoracic temperature required for flight, and consequently fall, crawl, and ultimately, die. Energy deficit could occur when bees continue to forage on linden despite limited nectar availability either due to loyalty to a previously energy-rich source or trigonelline-triggered memory/learning impairment, documented earlier with other alkaloids. Thus, the combination of low temperature and nectar volume, resource fidelity, and alkaloids in nectar could explain the unique phenomenon of bumble bee mortality associated with linden.

## Introduction

Bees provide critical pollination services in diverse landscapes, and are vital to global economy, food security, and environmental health. Worldwide, there have been reports of declines in bee populations due to loss of foraging resources and nesting habitats, pathogens, and pesticides [1–5]. Other factors may also be responsible for bee mortality but these have received little attention. Risks associated with foraging behaviors are particularly critical as bees spend considerable time seeking food resources. Social bees like honey bees and bumble bees depend on flowers from a diversity of plants and trees for colony survival, growth and reproduction [6,7]. Sugars, amino acids, lipids, vitamins, and minerals are obtained by bees from floral nectar and pollen to meet their metabolic needs [8–10], and thus, the availability of flowers that can provide these resources throughout the life cycle of the colony is critical.

Linden (*Tilia spp*.) is a profusely flowering urban tree that draws an abundance of bees during bloom [11,12]. Reaching heights of >20 meters, its broad crown offers dense flowers (2400 flowers/m^2^) with sugar rich nectar and high quality pollen for bees, making it and an attractive choice for urban developers and bees alike [13]. It has received considerable attention since 2013 when >50,000 bumble bee (*Bombus spp*.) workers were observed dead under blooming linden trees treated with pesticides in a parking lot [14]. Pesticides were clearly responsible for the bee deaths but the incident drew attention to earlier reports of bee mortality associated with linden. First highlighted in the 1970s, though referenced over a century ago [15], dead and crawling bumble bees have been observed beneath linden across Europe and North America, in numbers ranging from few to hundreds, and have involved multiple linden and bumble bee species [16–18]. Most linden associated bee mortality reports relate to bumble bees, and though a few dead honey bees have been observed, all references to bees in this paper pertain to bumble bee workers. Early explanations attributed bee mortality to the presence of mannose in nectar of linden trees under drought conditions [19]. Mannose, an isomer of glucose which is used by bees as a carbohydrate source, was speculated to disrupt glucose metabolism, resulting in toxicity to bees [20,21]. However, subsequent studies have refuted that mannose is a competitive inhibitor of glycolysis [22,23], and the presence of mannose in linden nectar has not been documented despite multiple analyses [24,25].

A second hypothesis suggests that bees die of starvation as a result of decreasing nectar production in late blooming linden [17,24]. Bloom in linden can last for several weeks but studies have shown that linden nectar production decreases as early as the second or third day [12,17,26]. It is speculated that, in areas where few other floral resources are available, bees forage until exhaustion on linden trees that had previously provided large nectar rewards because of the strong olfactory cues produced by flowers, and the tendency of bumble bees to show high resource fidelity [27–29]. Evidence for this hypothesis was provided by studies in which ‘dying’ bees collected under linden trees were shown to recover after being fed linden nectar [24,30]. The hypothesis contradicts evidence that bumble bee foraging behavior is highly calculated, involving the continual assessment of nectar rewards and associated energetic costs [28,31–34]. Moreover, many other plants offer little to no nectar reward and are not associated with bee mortality. Koch and Stevenson [34] review this hypothesis, among others, and ultimately conclude that starvation alone cannot fully explain the phenomenon as they observed >400 dead bees beneath a linden tree at the Royal Botanic Gardens, Kew, Richmond, UK, when alternative floral resources were present nearby. Thus, linden associated bee mortality remains a complex phenomenon, not yet adequately explained by mannose toxicity or by starvation alone.

Since the report of the massive bumble bee mortality in 2013, we have examined linden trees during bloom in various parts of western Oregon, USA. Annually, we have observed non-pesticide related mortality of the bumble bee species *B*. *vosnesenskii* associated with diverse linden species, namely *T*. *americana*, *T*. *cordata*, *T*. *platyphyllos* and *T*. *tomentosa*. We have noted that, at any given time, not every linden tree causes bee mortality, and that not every bee that forages on a linden tree dies. Also, linden trees that do not cause bee mortality during early bloom, do so during late bloom. Additionally, we have observed, as have other researchers [18,24], that bumble bee mortality under linden occurs when morning temperatures are below 30°C, the thoracic temperature required for flight in bees. Under these conditions, maintaining the necessary temperature for flight is energetically costly because bees must shiver their flight muscles to produce heat until they reach 30°C [35]. Thus, an energy deficit would lead to the bees dropping to the ground, crawling and ultimately dying. Energy deficit could occur when bees forage on linden flowers with low nectar. However, bees are not expected to seek low nectar flowers, given their ability to assess nectar rewards and associated energetic costs, unless they are drawn to low nectar flowers for other reasons. Bumble bees have been observed to exhibit fidelity to foraging resources [29]. It is also possible that compounds in nectar such as alkaloids could impact bee foraging behavior and result in bees foraging on low nectar flowers. Bees are drawn to the alkaloid nicotine which is speculated to be a source of self-medication against pathogen infections [36]. Nicotine, and the alkaloid caffeine, increase bee memory formation and learning [37,38]. If bees that have ingested alkaloids do not accurately reassess the tradeoff of nectar rewards and the energetic cost of foraging, they are at risk of an energy deficit. However, little is known about alkaloids in linden nectar.

Evidence of compounds present in nectar and evaluation of their impacts on bees are thus still needed for determining the basis of bee mortality associated with linden. The challenge lies in identification of multiple compounds in low volumes of nectar [39]. Nectar contains, besides sugars (sucrose, glucose, fructose), amino acids, proteins, lipids, fatty acids, phenolics, alkaloids, and organic acids [8,39]. Untargeted metabolic analysis, or fingerprinting, provides a powerful option as it can detect very small amounts of compounds including metabolic intermediates and secondary metabolites including alkaloids. Metabolomics has been used to compare nectars of two plant species in the same genus [40] and for understanding interactions between insects and their host plants [41]. Hence, metabolomic analysis of bee tissue and linden nectar could offer insight into the linden-bee mortality phenomenon first observed more than a century ago [15].

For insights on factors causing bee mortality we adopted a biochemical approach to investigate the metabolites and metabolic pathways associated with flight in bees. We conducted untargeted metabolomic analyses of thoracic muscle of healthy foragers and crawling bees observed beneath *T*. *cordata*, the linden species under which we observed the greatest number of crawling bees. Our objective was to show evidence of different metabolic states of healthy and crawling bees, and we hypothesized that metabolites involved in the production of energy for flight would be detected at higher levels in muscle of healthy bees than crawling bees. We also analyzed nectar of *T*. *cordata* to determine whether it contained alkaloids known to affect bee foraging behavior. A combination of methods was used for identification of a sufficiently broad range of metabolites for conducting pathway analysis, and for identification and quantification of sugars in nectar that are utilized in metabolism during flight.

## Materials and methods

### Sample preparation

#### Nectar

Nectar was collected from five trees under which dead bumble bees were observed. Trees were located at one of three sites, each containing several trees, around the Oregon State University campus in Corvallis, OR, USA. Samples were collected at various times of day using 0.5μl microcapillary pipettes. In 2016, eight samples were collected from one tree on two separate occasions, and in 2017 ten samples were collected from five trees over several days. Nectar was extracted from microcapillary pipettes by homogenizing the nectar-filled capillaries in bead blast tubes in a 50:50 mixture of methanol and water. Microcapillaries were pulverized with microbeads and separated from the nectar solution by centrifugation for 13 minutes at 15,000 x g at 4°C. Supernatant was removed and stored in glass vials to at -80°C until analysis.

#### Bee muscle

Bees were collected on and beneath *T*. *cordata* trees at the same three sites around the Oregon State University Campus located in Corvallis, Oregon, USA. Bee collection occurred over the same 7–9 day time period when flowers were in bloom that nectar collection occurred. All bees analyzed in this study were wild caught bees, therefore colony characteristics are not known. To ensure crawling bees were not simply nearing the end of their natural lifespan, those with visibly tattered wings indicative of old age were not selected for analysis. Healthy bees were actively foraging when captured. Crawling bees were collected as they were encountered, therefore the length of time between when they fell and when they were captured by us was often unknown. However, based on how the sites were sampled (walking back and forth beneath rows of trees), we estimate that roughly one third of crawling bees captured in 2017 were caught within five minutes of falling from a linden tree. It was also not possible to identify the exact tree within a site from which crawling bees fell because they often crawl quickly in the first minutes after falling and the crowns of neighboring trees often overlapped. In 2016, 10 crawling and 9 healthy bees were captured, immediately placed in a cooler, and transported to the lab where muscles were removed and placed in liquid nitrogen. In 2017, 19 crawling bees and 19 healthy bees were caught, placed on dry ice, and stored in a freezer until muscles were removed at a later date. After removal from bees, muscles were stored in vials in an -80°C freezer until analysis by liquid chromatography-mass spectrometry (LC-MS) and nuclear magnetic resonance (NMR).

### Chemical analysis of nectar and muscle

#### Untargeted LC-MS/MS

High pressure liquid chromatography of nectar and muscle was performed on a Shimadzu Nexera system (Shimadzu, Columbia, MD, USA) coupled to a hybrid quadrupole-time of flight mass spectrometer (TripleTOF^TM^ 5600, AB SCIEX). Raw LC-MS/MS data files were imported into MarkerView software (AB SCIEX) for initial data processing including feature detection, peak alignment, peak integration, and principal component analysis. A portion of metabolites was identified using the Mass Spectrometry Metabolite Library of Standards library (IROA Technologies, LLC), which contains >600 metabolites. Details of LC-MS/MS analysis can be found in the supplementary files (S1 Appendix).

#### Nuclear magnetic resonance

A subset of muscle samples (healthy *n* = 4, crawling *n* = 5) and nectar (*n* = 6) was randomly selected from those collected in 2017, previously analyzed with LC-MS/MS, to be analyzed by nuclear magnetic resonance (NMR) to identify and quantify several sugars that could not be distinguished by LC-MS/MS without complex derivatization and to confirm the presence of other detected compounds. NMR analysis was performed on an 800 MHz Bruker Avance III HD NMR spectrometer equipped with 5mm cryogenic triple resonance (HCN) probe. Details can be found in supplementary files (S1 Appendix). Data were processed, apodized, phased and spline baseline corrected using the Chenomx software suite (Edmonton, Canada). Metabolite profiling was performed manually in the Chenomx software suite.

#### Targeted trigonelline quantification

A targeted search for trigonelline in linden nectar was conducted with remaining nectar samples collected in 2017 after LC-MS/MS and NMR analysis. A trigonelline standard (TCI America, Portland, Oregon) was used for calibration curves and instrument optimization. Analytical separations were performed on a Shimadzu HPLC (Shimadzu, Columbia, MD) with a 4.6 × 150 mm, 5 μm, Inertsil Phenyl-3 HPLC column (GL Sciences, Japan). Details can be found in supplementary files (S1 Appendix)

### Statistical analysis

Thoracic muscle metabolites of healthy and crawling bees identified with LC-MS/MS were analyzed using SAS version 9.4. Group differences were calculated overall and separately for data from 2016 and 2017 using a parametric t-test and a non-parametric Wilcoxon Rank Sum test. *Q*-values were calculated from *P*-values using the Benjamini Hochberg False Discovery Rate (FDR) procedure, a less conservative correction for multiple comparisons than the Bonferroni correction. All tests were two-sided. Significance was set at 0.05. Supervised principal component analysis was conducted in MarkerView 1.2 using Pareto scaling, similar to autoscaling but with stronger reduction in the impact of metabolites with large fold changes. Initial principal component analysis included quality control samples (QCs) and indicated that variation due to instrumentation was minimal and hence, QCs were removed from subsequent analysis (S1 Fig).

Metabolites detected by NMR were analyzed in Microsoft Excel 2018 and in R version 3.5.3 (©2019). Prior to analysis, data were tested for normality using the Shapiro-Wilk test and homoscedasticity using an F-test. Not all variances were equal, therefore we used Welch’s unequal variance t-tests to compare metabolites in healthy and crawling bees, and *Q*-values were calculated from *P*-values using the Benjamini Hochberg FDR procedure. Detection of two metabolites, trehalose and sucrose, was not sufficient to conduct a t-test so the Fisher’s exact test (detectable vs. nondetectable) was done. All tests were two-sided, and significance was set at *𝛼* = 0.05.

#### Pathway analysis

Pathway analysis was conducted with MetaboAnalyst 4.0 on thirty-four metabolites that differed significantly between healthy and crawling bee muscle. The *Drosophila melanogaster* pathway library was used, and overrepresentation analysis was accomplished with the hypergeometric test. *P*-values were calculated with enrichment analysis, and these were adjusted using False Discovery Rate. For isomers that could not be fully distinguished with LC-MS/MS, one isomer was selected for pathway analysis provided it did not affect the statistical outcome (citrate/isocitrate, threonine/allothreonine/homoserine). One metabolite, L-oleoyl-rac-glycerol, could not be identified by the program and was subsequently excluded from analysis.

#### Heatmap analysis

Heatmap analysis was conducted in MetaboAnalyst 4.0 using features determined to be significantly different between muscle of healthy and crawling bees. Due to differences between years likely resulting from differences in muscle collection and storage methods, the heatmap was constructed using only data from 2017, the year with larger sample sizes. The heatmap was constructed using the Euclidian distance measure and Ward clustering algorithm.

## Results

### Bee muscle

In the thoracic muscles of healthy and crawling bees collected from linden trees in 2016 and 2017, one hundred and fifteen features detected with liquid chromatography–tandem mass spectrometry (LC-MS/MS) matched records in the IROA library of metabolite standards. Metabolites belonged to different classes of compounds, including purines (hypoxanthine, xanthine), amino acids (lysine, tyrosine, histidine, proline, serine, aspartate), organic acids (fumarate, malate, palmitate), and many others (S1 Table). A non-parametric Wilcoxon Rank Sum test revealed that thirty-two metabolites differed significantly in peak intensity between muscle from healthy and crawling bees (Table 1; *P* < 0.05).

**Table 1:** Comparison of peak intensity of metabolites identified by LC-MS/MS between thoracic muscle of crawling (n = 29) and healthy (n = 28) bees collected on or beneath *T*. *cordata* in 2016 and 2017.

Metabolite*	Ratio^†^ (Crawlers:Healthy)	*P*	*Q*
*Energy Metabolism*:			
Citrate/isocitrate	0.35	< .0001	< .0001^‡^
Ketoglutaric acid	0.41	< .0001	< .0001^‡^
Malate	0.42	< .0001	0.0002^‡^
(S)-lactate	0.44	0.0004	0.005^‡^
Fumarate	0.49	0.001	0.01^‡^
N-acetyl-d-mannosamine	0.46	0.008	0.04^‡^
Succinate	0.58	0.02	0.11
*Nucleotide Metabolism*:			
Cytosine	0.46	< .0001	0.001^‡^
Theophylline	2.24^†^	0.0004	0.005^‡^
Allantoin	0.51	0.0006	0.005^‡^
Adenosine	0.58	0.001	0.01^‡^
Hypoxanthine	0.52	0.005	0.03^‡^
Xanthine	0.68	0.01	0.07
Guanosine 5'-diphosphate	1.72^†^	0.02	0.11
Inosine	2.06^†^	0.05	0.16
*Amino Acid Metabolism*:			
Tyrosine	0.38	< .0001	< .0001^‡^
N-acetyl-l-phenylalanine	0.46	0.0001	0.002^‡^
Nε,Nε,Nε-trimethyllysine	0.49	0.0002	0.002^‡^
Lysine	0.51	0.0002	0.003^‡^
Cadaverine	0.56	0.002	0.02^‡^
N(pai)-methyl-l-histidine	0.63	0.006	0.03^‡^
Putrescine	0.64	0.02	0.09
Tryptophan	0.60	0.03	0.11
Proline	0.63	0.03	0.11
Shikimate	1.78^†^	0.02	0.11
4-aminobutanoic acid	1.77^†^	0.03	0.11
Pipecolic acid	0.57	0.03	0.13
Glutamine	0.63	0.04	0.16
Threonine/allothreonine/homoserine	0.70	0.04	0.16
*Lipid Metabolism*:			
Lauric acid	0.47	< .0001	0.001^‡^
Phosphocholine chloride	0.53	0.002	0.02^‡^
L-oleoyl-rac-glycerol	0.48	0.003	0.02^‡^

* metabolites with *P* < 0.05 after comparison using a non-parametric Wilcoxon Rank Sum test

† indicates ratios >1 in which median peak intensity was higher in muscle of crawling bees than healthy bees

‡ indicates metabolites that were significant after controlling the false discovery rate (Benjamini Hochburg Procedure; *Q* < 0.05)

**Fig 1:**
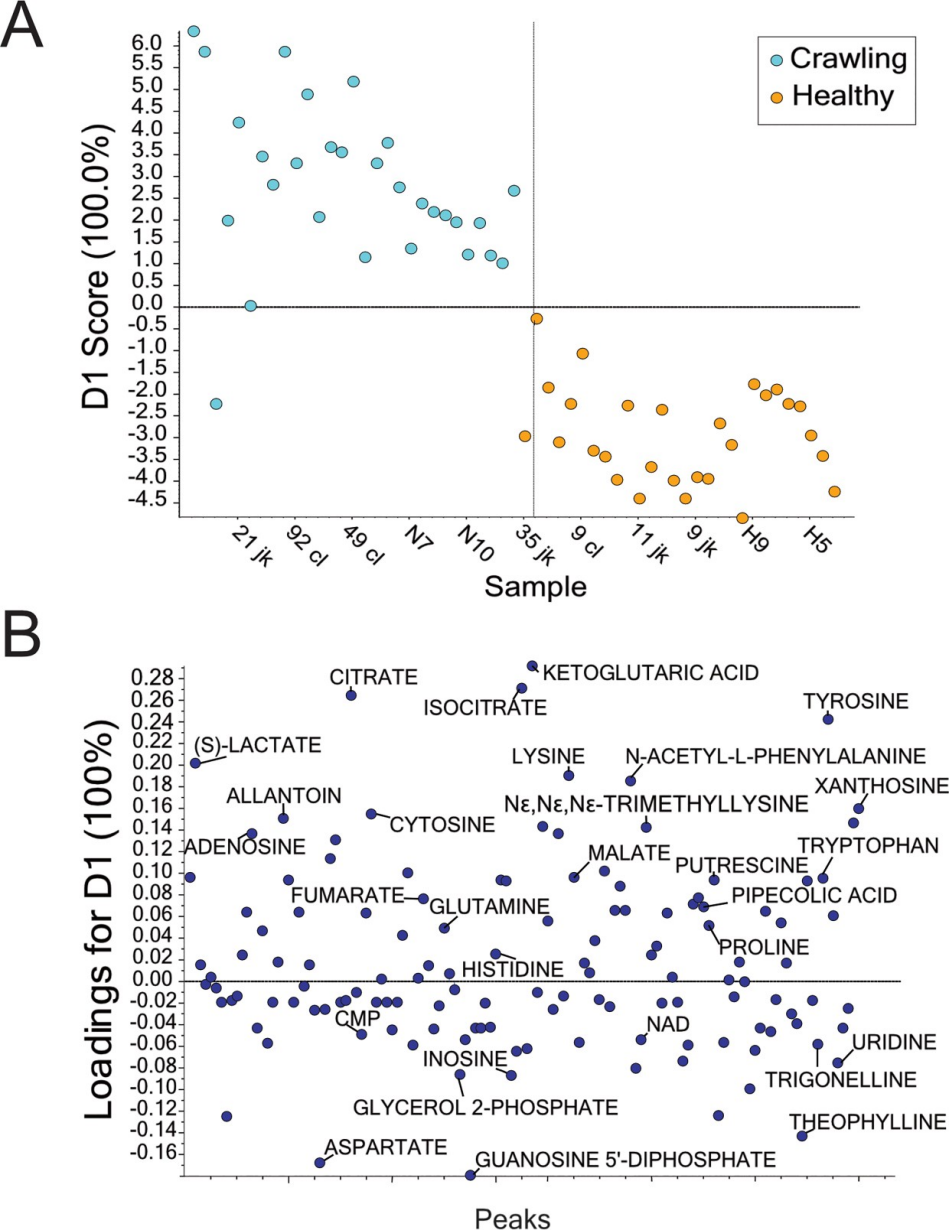
Results of supervised principal component analysis of metabolites detected by LC-MS/MS in bee muscle. Muscle was collected from 28 healthy and 29 crawling bees over two years (2016 and 2017). *(A)* Scores plot. Data were log transformed and Pareto scaling was used to account for differences in peak magnitude and minimize the noise of small variables. *(B)* Loadings plot. The location of each metabolite in relation to 0 indicates the strength of its contribution on the principal component: metabolites further from 0 contributed more than those closer to 0, and metabolites above 0 were associated with crawling bees whereas those below 0 were associated with healthy bees. Metabolites are arranged along the x-axis according to retention time.

The supervised principal component analysis (PCA) comparing metabolic state of the thoracic muscle of healthy and crawling bees showed clear separation between groups (Fig 1A). The loadings plot (Fig 1B) illustrated the strength of contribution of each variable (metabolite) to the principal component, indicated by its relative distance from zero.

Analysis of muscle samples with nuclear magnetic resonance (NMR) confirmed the presence of 14 metabolites and identified 8 additional metabolites that were not detected with LC-MS/MS (Table 2). Two metabolites, β-alanine and formate, detected with NMR but not detected with LC-MS/MS, varied significantly between healthy and crawling bees (Welch’s t-test; *β*-alanine *Q* = 0.03; formate *Q =* 0.02), with formate levels higher in crawling bees and *β*-alanine higher in healthy bees. The concentration of carbohydrates glucose and fructose did not differ between healthy and crawling bee muscle. Trehalose was detected only once in healthy bees, and in 4 of the 5 crawling bees analyzed, and the difference in trehalose detection rate was not significant (Fisher’s exact test; *P* = 0.21). However, trehalose may have been present at levels below the NMR lower detection limit, therefore further analysis with equipment capable of detecting very small quantities of this important sugar is necessary.

**Table 2:** Comparison of metabolites detected by NMR in thoracic muscles of crawling and healthy bees collected on or beneath linden in 2017.

Metabolite	N*	Ratio^†^	*P*	*Q*
*Amino Acid Metabolism*				
Formate	5c 4h	1.32^†^	0.02	0.02^§^
*β*-Alanine	5c 4h	0.57	0.02	0.03^§^
Glutamate	5c 4h	0.64	0.16	0.20
Leucine	5c 4h	1.46^†^	0.20	0.27
Taurine	5c 4h	0.82	0.21	0.33
Pyroglutamate	3c 2h	0.77	0.39	0.72
Glutamine	5c 4h	1.19^†^	0.59	1.17
Proline	5c 2h	1.10^†^	0.82	2.34
Alanine	5c 4h	0.97	0.86	2.86
Sarcosine	5c 4h	1.05^†^	0.88	4.38
Valine	5c 4h	1.01^†^	0.97	9.70
Ethanolamine	5c 4h	1.01^†^	0.98	19.56
*Energy Metabolism*				
Lactate	5c 4h	5.72^†^	0.14	0.17
Succinate	3c 3h	3.20^†^	0.21	0.30
Glucose	5c 4h	1.38^†^	0.38	0.63
Fructose	5c 4h	1.29^†^	0.64	1.43
Trehalose	4c 1h	12.71^†^	0.21^‡^	-
Sucrose	1c 1h	7.59^†^	1.00^‡^	-
*Lipid Metabolism*				
Choline	5c 4h	1.13^†^	0.69	1.73
sn-Glycero-3-phosphocholine	5c 4h	0.96	0.87	3.46
O-Phosphocholine	5c 4h	0.96	0.89	5.94
*Nucleotide Metabolism*				
Inosine	5c 4h	0.57	0.003	0.003^§^

* Represents the numbers of crawlers (c) and healthy (h) bees analyzed, a subset of those analyzed with LC-MS/MS in 2017. Some metabolites were not detected in all samples, therefore *n* is lower.

† indicates ratios >1 in which mean concentration was higher in muscle of crawling bees than healthy bees.

‡ *P*-value based on Fisher’s exact test (detectable vs. non-detectable), as four of five crawling but only one of four healthy bees had detectable levels of trehalose.

§ indicates significance at α = 0.05

**Fig 2:**
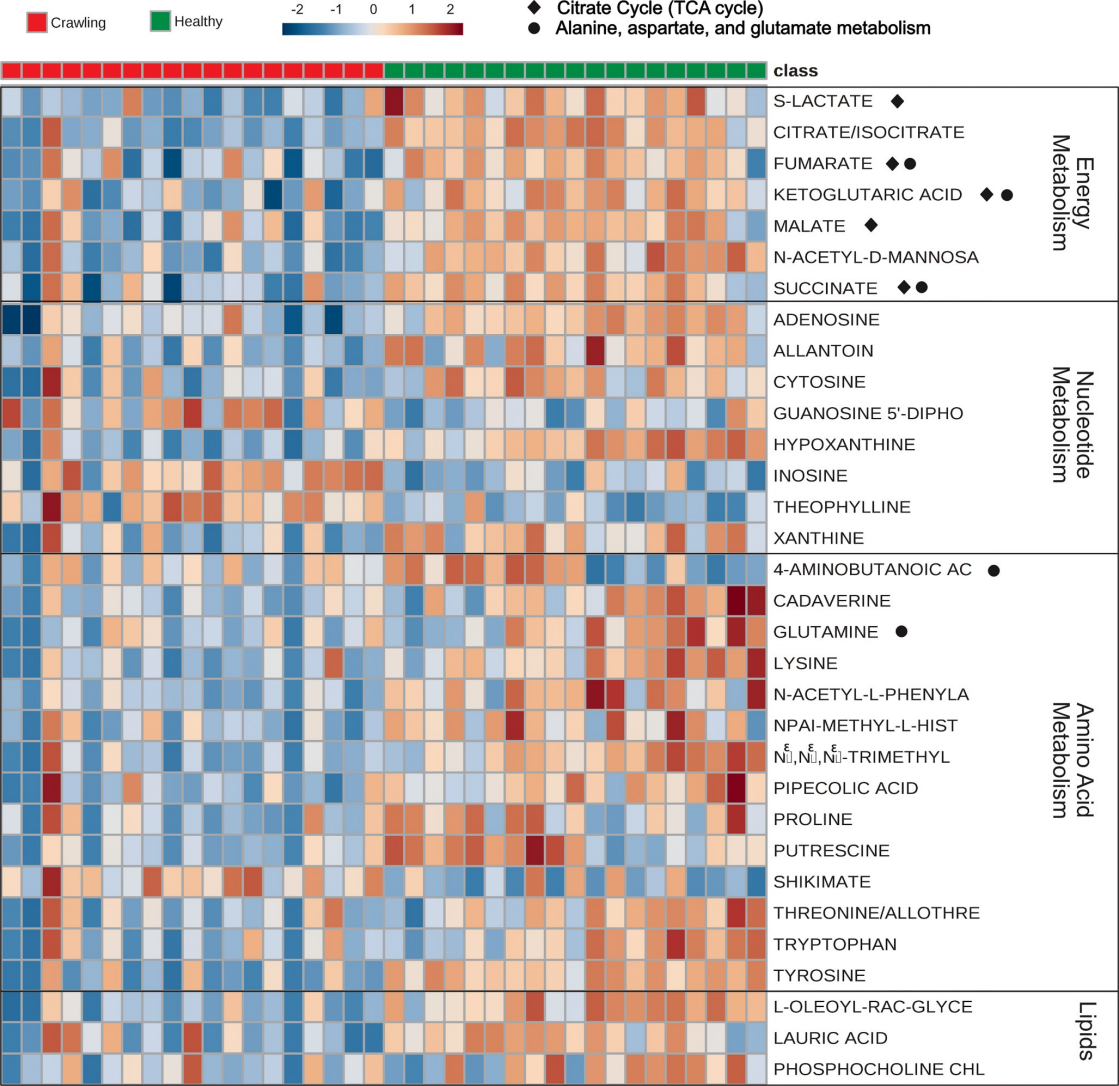
Heat map of thirty-two metabolite profiles detected with LC-MS/MS in muscle of 19 healthy and 19 crawling bees collected in 2017. Features with *P* > 0.05 and metabolites detected with NMR were excluded for ease of comparison. The heat map depicts relative levels of metabolites, determined with Pareto scaling, that range from high (+2; red) to low (-2; blue) in healthy and crawling bee muscle. Metabolites are grouped according to their role in prominent pathways.

Pathway analysis conducted with the 34 metabolites that differed significantly between thoracic muscle of healthy and crawling bees identified two metabolic pathways with constituents present significantly more frequently than expected at random (Table 3). These were the TCA cycle in which five of the twenty total metabolites were represented (ketoglutaric acid, succinic acid, malic acid, citric acid, fumaric acid), and alanine-aspartate-glutamate metabolism in which five of twenty-three total metabolites were represented (ketoglutaric acid, glutamine, 4-aminobutanoic acid, fumaric acid, succinic acid). The remaining compounds in each pathway were either not detected or were not significantly different between muscle of healthy and crawling bees. Metabolites involved in the metabolism of purine, glyoxylate and dicarboxylate, and several other amino acids were strongly represented in pathway analysis but were not present significantly more frequently than expected at random. Heat map analysis also indicated that all metabolites involved in the TCA cycle were present at higher levels in healthy bee muscle compared to crawling bee muscle (Fig 2).

**Table 3:** Major metabolic pathways identified by pathway analysis. Analysis included thirty-three metabolites detected by LC-MS/MS and NMR that differed significantly between the muscles of crawling and healthy bees. The raw *P*-value was generated from enrichment analysis alone and the False Discovery Rate (FDR) value was calculated to adjust for multiple testing.

Pathway Name	Metabolites detected/Total metabolites in pathway	*P*	FDR
Citrate cycle (TCA cycle)	5/20	0.00063	0.02*
Alanine, aspartate and glutamate metabolism	5/23	0.0006	0.02*
Arginine and proline metabolism	5/37	0.006	0.15
D-Glutamine and D-glutamate metabolism	2/5	0.01	0.19
Glyoxylate and dicarboxylate metabolism	3/16	0.01	0.19
Purine metabolism	6/64	0.01	0.19

* indicates significance at *α* < 0.05

### Nectar

**Fig 3:**
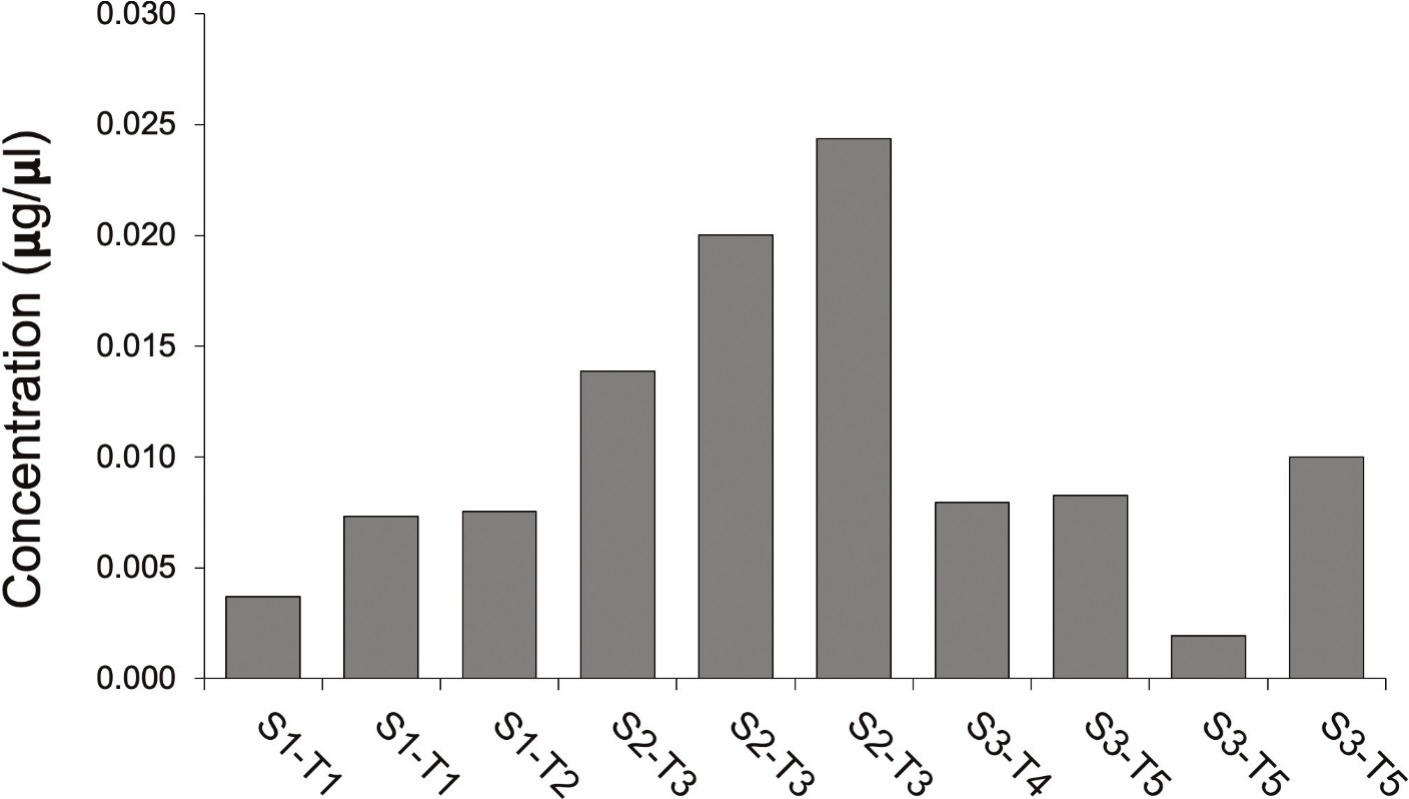
Trigonelline concentration in nectar from linden flowers collected in 2017. Samples were collected from individual flowers from five trees known to kill bees spanning three sites and an eight-day period during which crawling bees were found. Some trees were sampled more than once. (S = Site; T = Tree).

Ninety-four features in *T*. *cordata* nectar detected with LC-MS/MS matched records in the IROA library of biochemicals (S2 Table). Most notably, the alkaloids caffeine and trigonelline were detected in *T*. *cordata* nectar. In our study, caffeine was detected in samples collected in 2016, and trigonelline was detected in both 2016 and 2017 samples. Since trigonelline is a pyridine alkaloid, and the presence of the related alkaloid nicotine in nectar is known to impact bee foraging behavior, we conducted additional targeted analysis of samples collected in 2017. This analysis revealed trigonelline quantities ranging from 1.94 ng/ml to 2.44 ng/ml (Fig 3), a much lower level than other alkaloids that have been detected in nectar but one that could be biologically active [37].

Nuclear magnetic resonance identified and quantified 8 metabolites in the subset of 2017 nectar samples analyzed, including four metabolites not identified by LC-MS/MS, and sugars fructose, glucose, and sucrose (Table 4). Like many nectars, *T*. *cordata* nectar is sucrose dominant (0.577 mM) but also contains both glucose (0.243 mM) and fructose (0.291 mM). Trigonelline and caffeine were detected by LC-MS/MS but not by NMR which has a higher (1 mM) detection limit. Mannose was not detected by LC-MS/MS or NMR, once again confirming its absence in linden nectar.

**Table 4:** Metabolites detected by NMR in linden nectar in 2017.

Metabolite	*N*	Concentration (mM)
*Alcohol*:		
Ethanol	5	0.003
Isopropanol	5	0.008
Methanol	6	0.03
*Carbohydrate*:		
Fructose	6	0.29
Sucrose	6	0.58
Lactate	6	0.01
Glucose	6	0.24
*Carboxylic Acid*:		
Acetate	6	0.005

Samples represent a subset of those analyzed with LC-MS/MS in 2017 (*n* = 6). Some metabolites were not detected in all samples, therefore *n* is lower.

## Discussion

This is the first study that has examined metabolites and metabolic pathways in search of insights on the linden associated mortality of bumble bees. With untargeted metabolomic analysis, principal component analysis, and pathway analysis, we determined that the metabolic state is very different between muscles of healthy bees and crawling bees associated with linden. Many metabolites that differed between healthy and crawling bees are associated with energy production, and thus provide evidence that energy deficiency is a factor. This is also the first study to document the presence of the alkaloid trigonelline in the nectar of linden. This is significant as alkaloids have been shown to influence bee foraging behavior by impacting memory formation [37,38], resulting in suboptimal foraging choices. The energy deficiency documented here, and factors including the presence of trigonelline that could be linked to it, offer an explanation for the mortality of bumble bees foraging on linden that has been observed in Europe and North America since over a century ago.

In this study, we identified 123 metabolites in bee thoracic muscles, of which more than 25 percent differed in peak intensity between healthy and crawling bees. By using principal component analysis and pathway analysis we were able to narrow our focus to a handful of metabolic pathways that could be impacted in bees that forage on linden. Both heatmap and pathway analyses point to involvement of the TCA cycle which is the primary link to oxidative phosphorylation and the production of ATP that powers muscle contraction. The presence of lower levels of five TCA metabolites (ketoglutaric acid, succinic acid, malic acid, citric acid, fumaric acid) in crawling bees indicates that the central metabolism is affected. Carbohydrates from nectar enter the TCA cycle and pass through several intermediates including those we detected (citrate/isocitrate, fumarate, succinate), releasing NADH that results in ATP production; hence a reduction in these intermediates in crawling bees is consistent with a carbohydrate shortage.

An average foraging bee expends 0.2–0.5 calories/minute while foraging, a rate double that of active vertebrates, including hummingbirds, and one that is demanding to maintain with little fuel reserves [35,42]. Our observation that higher numbers of crawling bees were found in the morning, when temperatures are cooler (<30°C), aligns with the hypothesis that bees experience an energy shortage, as cool morning temperatures require significant energy expenditure for thermoregulation alone. Late blooming linden trees with low nectar volume [12] may not provide the caloric requirements for both flight and thermoregulation. Bees that continue to forage despite cool temperatures and low nectar volume in flowers are thus at risk of starvation.

Metabolites involved in alanine, aspartate, and glutamate metabolism were strongly represented in pathway analysis in our study, likely because the synthesis of these amino acids is closely linked to the TCA cycle (the pathway identified as most impacted), via transamination from TCA intermediates pyruvate, oxaloacetate, and α-ketoglutaric acid (via citrate), respectively. Other metabolites that differed significantly between healthy and crawling bee muscle are involved in purine metabolism. The nucleoside inosine was one of few metabolites detected at higher levels in crawlers than in healthy bees, and this, paired with lower levels of metabolic intermediates hypoxanthine and xanthine, suggests that it is being produced in response to energy stress in crawling bees. Inosine plays a critical role in the purine nucleotide cycle. Its production in mammalian skeletal muscle facilitates the regeneration of ATP from ADP by removing the byproduct AMP through its conversion into inosine [43]. In zebrafish muscle under energy stress, inosine functions as a sink for AMP, which is produced in the myokinase reaction (2 ADP → ATP + AMP) to recover ATP from ADP [44].

Sugars fructose and glucose levels did not differ between muscles of healthy and crawling bees in the subset analyzed with NMR, and trehalose was detected frequently in crawling bees but remained below the detection limit in all but one healthy bee. We suspect that low trehalose levels in healthy bees reflect a higher metabolic rate in which trehalose is rapidly broken down to supply glucose molecules, compared with that of crawling bees in which the reverse occurrs–trehalose is metabolized at a slower rate and therefore higher levels are present. Why glucose and fructose did not differ is unknown; we speculate that their production is closely tied to metabolic rate. For instance, if glucose remains locked in the disaccharide trehalose until it is needed in metabolism, a large buildup may not occur even as metabolism slows down.

Linden nectar was found to contain levels of fructose, glucose, and sucrose similar to previous studies [45] and to other bee-attractive flowers [46]. It also contained other compounds including amino acids, alkaloids and others. The alkaloid trigonelline has been identified previously in honey samples from Europe and South America, including monofloral *Coffea* honey that also contained high levels of caffeine [47, 48], but this is the first study in which trigonelline has been identified in nectar. We hypothesize that it, alone or in combination with other alkaloids, influences floral constancy of foragers, resulting in bees continuing to forage on linden flowers despite a reduction in nectar production late in the blooming period. Trigonelline has been postulated to have weak cholinesterase inhibitory activity [49], reducing the breakdown of the excitatory neurotransmitter acetylcholine involved in learning and memory in mammals and insects [50,51]. Similar neurological mechanisms are affected by other alkaloids which have been shown to result in enhanced flower constancy to flowers with suboptimal nectar rewards in choice experiments performed in a lab [38]. Caffeine increases the activation of nicotinic acetylcholine receptors by lowering the action potential threshold in the Kenyon Cells, important in associative learning[37], thus increasing the likelihood of action potentials firing when a stimulus is present. Nicotine is speculated to increase the neurons receiving acetylcholine at their synapses; similar to caffeine, it increases associative learning in bees [38]. We detected lower trigonelline levels (ng/ml) than those of caffeine and nicotine (μg/ml) with documented effects on bees. However, small quantities can have physiological effects, and the lowest quantity at which bee foraging is affected has not been determined.

If trigonelline affects bee learning and memory as has been demonstrated for caffeine and nicotine, skewed resource loyalty could be a factor in the linden-bee mortality phenomenon. Bumble bees that experience a high nectar reward during early bloom may develop a strong loyalty to it and continue to return throughout bloom, even when nectar volume is low. Volatiles from linden nectar brought by foragers and stored in honey pots and circulating in nest air provide cues to other bees in the colony about good floral resources [52–55]; therefore, as linden volatiles increase in a nest, an increasing numbers of bumble bee foragers receive cues to seek out linden trees. Bumble bees have the ability to assess nectar rewards and should thus avoid flowers with low nectar, but if bees follow cues from within the nest to a nectar source and experience strong associative learning due to alkaloids in that nectar, they may continue to foraging on flowers even if nectar levels become suboptimal.

Other alkaloids that could contribute to bee mortality could be present in linden nectar but not yet detected, as even detection of common alkaloids nicotine and caffeine has been inconsistent. Our untargeted LC-MS/MS analysis of nectar did not detect nicotine, and detected caffeine in samples analyzed in only one of the two years of the study. A recent targeted GC-MS search by Jacquemart et al. [45] for nicotine in linden nectar did not detect it. However, in a study by Naef *et al*. [56] trace amounts of both nicotine and caffeine were detected. It is possible that production of alkaloids varies among individuals; it may be temporal or an induced response to factors not yet determined.

## Conclusion

The metabolomic approach adopted in this study revealed that crawling bees experience an energy deficit compared with healthy bees foraging on linden, and that linden nectar contains the alkaloid trigonelline. Based on these findings, we propose the following explanation for bee mortality beneath linden: when ambient temperature is below 30°C, bees with an energy deficit that cannot maintain the necessary thoracic temperature for flight drop to the ground, crawl, and ultimately die of starvation. Energy deficit could occur when bees continue to forage on linden despite limited nectar availability either due to loyalty to a previously energy-rich source or impairment in memory/learning after exposure to trigonelline in nectar, as has been documented with other alkaloids. Thus, the combination of low temperature and nectar volume, resource fidelity, and alkaloids in nectar could explain the unique phenomenon of bumble bee mortality associated with linden. This requisite combination of factors aligns with aspects of the phenomenon that we have observed (indicated earlier), that at any given time not every linden tree causes bee mortality and not every bee that forages on a linden tree dies, and linden trees that do not cause bee mortality during early bloom do so during late bloom. It is possible that other floral resources could meet these conditions and result in similar bee mortality, though none have been documented. Further studies are needed to directly link the presence of trigonelline in nectar with the mechanisms that would result in suboptimal foraging choices in bumble bees.

## Supporting information

S1 Fig Results of supervised principal component analysis of metabolites detected by LC-MS/MS in bee muscle. Muscle was collected from 28 healthy and 29 crawling bees over two years (2016 and 2017). Quality control (QC) samples are tightly clustered indicating that system variance is negligible.
(TIFF)

S1 Table Comparison of all metabolites detected by LC-MS/MS between thoracic muscle of crawling and healthy bees collected on or beneath *T. cordata* in 2016 and 2017. (XLSX)

S2 Table Metabolites detected using LC-MS/MS in nectar collected from *T. cordata*. (XLSX)

S1 Appendix Protocols for analysis with targeted and untargeted liquid chromatography and nuclear magnetic resonance. (DOCX)
