# Supplementary figures and images for: Linden (Tilia cordata) associated bumble bee mortality: Metabolomic analysis of nectar and bee muscle
Source: PLoS One. 2019 Jul 10;14(7):e0218406. doi: 10.1371/journal.pone.0218406 (PMC6619659; doi:10.1371/journal.pone.0218406)

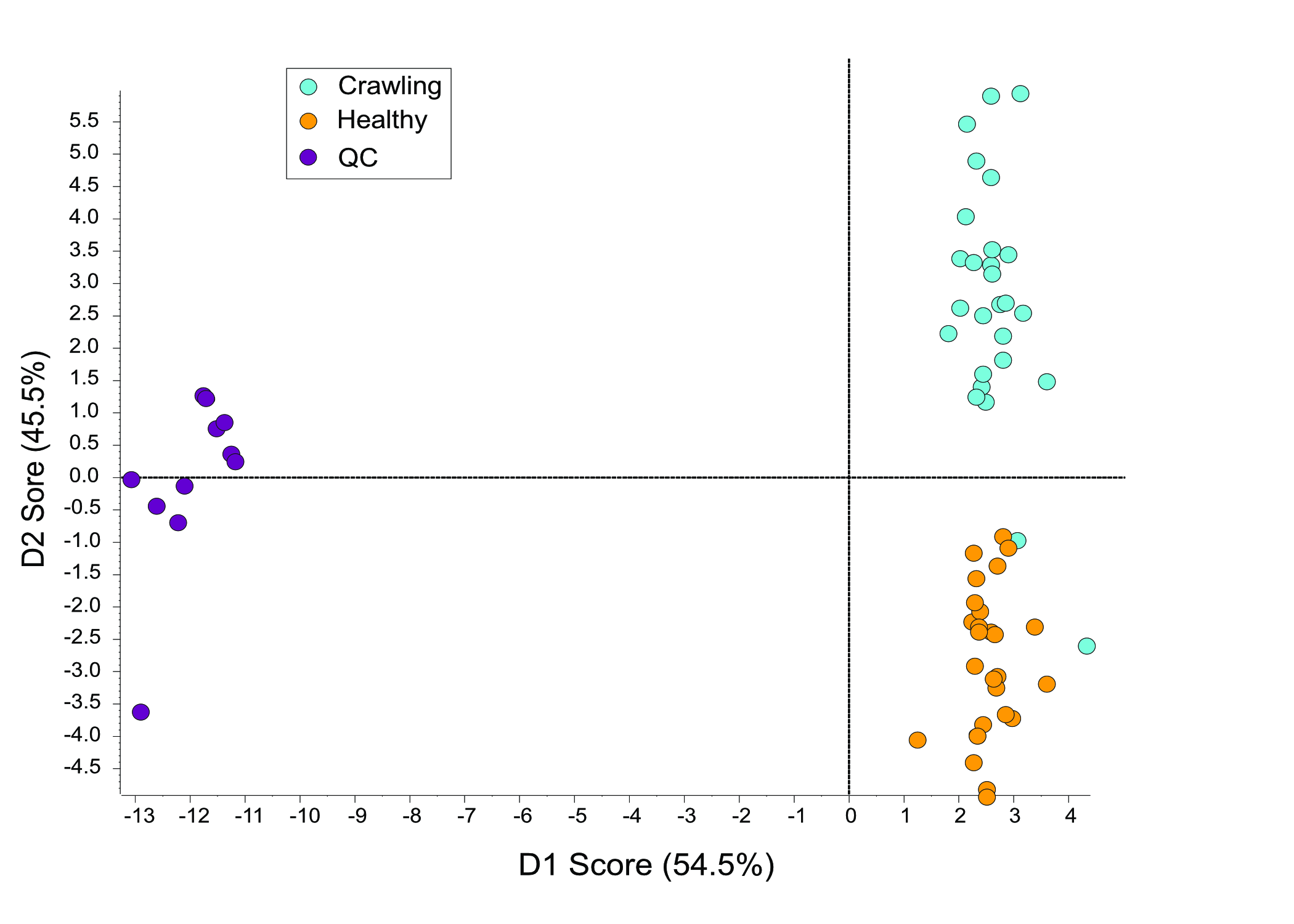

Supplement: S1 Fig — Muscle was collected from 28 healthy and 29 crawling bees over two years (2016 and 2017). Quality control (QC) samples are tightly clustered indicating that system variance is negligible. (TIFF) [file pone.0218406.s001.tiff]
