# Supplementary material for: Linden (Tilia cordata) associated bumble bee mortality: Metabolomic analysis of nectar and bee muscle
Source: PLoS One. 2019 Jul 10;14(7):e0218406. doi: 10.1371/journal.pone.0218406 (PMC6619659; doi:10.1371/journal.pone.0218406)
Supplement: S1 Appendix — (DOCX) [file pone.0218406.s004.docx]

**S1 File. Protocols for analysis with targeted and untargeted liquid chromatography and nuclear magnetic resonance.**

**Untargeted LC-MS/MS**

High pressure liquid chromatography of nectar and muscle were performed on a Shimadzu Nexera system (Shimadzu, Columbia, MD, USA) coupled to a hybrid quadrupole-time of flight mass spectrometer (TripleTOF^TM^ 5600, AB SCIEX). Chromatographic separations were carried out on an Inertsil Phenyl-3 column (150 × 4.6 mm, 5 μm; MetaChem Technologies, Torrance, CA) using a flow rate of 0.4 mL/min and mobile phases consisting of water (A) methanol (B), and both with 0.1% formic acid. The elution gradient was as follows: 0 min, 5% B; 1 min, 5% B; 11 min, 30 % B; 23 min, 100% B; 35 min, 100% B; 37 min, 5% B; 47 min, 5% B. The temperature of the column was held at 50°C and the injection volume was 10 µl.

Mass spectrometry analyses were performed on an AB SCIEX TripleTOF^TM^ 5600 equipped with an electrospray ionization source. The instrument was operated in the information dependent MS/MS acquisition mode with the collision energy set at 35 V and with a collision energy spread of 15 V. The scan range was *m/z* 70-1250 for ToF MS and *m/z* 50-1250 for MS/MS. For quality control and to determine system variance relative to biological variance, a mixture of equal aliquots of all samples was created and injected periodically throughout the experimental tests. Two-minute auto-calibrations were performed on every three samples. For samples analyzed from 2016 and 2017, three and eight quality controls were tested, respectively.

Raw LC-MS/MS data files were imported into MarkerView software (AB SCIEX) for initial data processing including feature detection, peak alignment, peak integration, and principal component analysis. A feature was defined as any *m/z* value detected at a unique retention time. A portion of metabolites was identified using the Mass Spectrometry Metabolite Library of Standards library (IROA Technologies, LLC), which contains >600 metabolites.

**Trigonelline Quantification**

A trigonelline standard (TCI America, Portland, Oregon) was used for calibration curves and instrument optimization. Analytical separations were performed on a Shimadzu HPLC (Shimadzu, Columbia, MD) with a 4.6 × 150 mm, 5 μm, Inertsil Phenyl-3 HPLC column (GL Sciences, Japan). The gradient was the same as described above to maintain similar retention times and ionization. A standard curve of 1, 5, 10, 50, and 100 ng/ml was prepared in a 50:50 mixture of water and methanol by volume. The mass spectrometry analysis was performed on an Applied Biosystems 4000 Qtrap (AB SCIEX, Foster City, CA) in positive MRM (multiple reaction monitoring) mode. The following MRM transition was used for quantification at retention time 10.7 minutes: *m/z* 138.2 → 94.0. The MRM transitions *m/z* 138.2 → 92.0 and *m/z* 138.2 → 78.0 were used as qualifiers. Tests for matrix effects were carried out and were determined to be negligible.

**Nuclear Magnetic Resonance**

A subset of muscle samples (healthy *n* = 4, crawling *n* = 5) and nectar (*n* = 6) was randomly selected from those collected in 2017 and previously analyzed with LC-MS/MS, and analyzed on an 800 MHz Bruker Avance III HD nuclear magnetic resonance (NMR) spectrometer equipped with 5mm cryogenic triple resonance (HCN) probe. Samples for NMR were dried and dissolved in 200 µl of 10% D_2_O, 90% H_2_O, supplemented with 0.511 mM DSS and 0.01% sodium azide and loaded into 3mm NMR tubes. The ^1^H experiment samples were collected using the recommended parameters for the Chenomx software to enable accurate concentration estimates. A 1D NOESY pulse sequence, with presaturation for water suppression, was used with a spectral window of 12 ppm, 4 second (s) acquisition time, 1 s recycle delay, and a total of 512 scans per experiment. All data were collected at 25° C. Data were processed, apodized, phased and spline baseline corrected using the Chenomx software suite (Edmonton, Canada). Metabolite profiling was performed manually in the Chenomx software suite.
